# Radon Exposure Assessment in Occupational and Environmental Settings: An Overview of Instruments and Methods

**DOI:** 10.3390/s24102966

**Published:** 2024-05-07

**Authors:** Mota Kholopo, Phoka Caiphus Rathebe

**Affiliations:** Department of Environmental Health, Faculty of Health Sciences, Doornfontein Campus, University of Johannesburg, P.O. Box 524, Johannesburg 2006, South Africa; motaliok@gmail.com

**Keywords:** radon, environmental exposure, occupational setting, monitoring, assessment strategies

## Abstract

Radon is a naturally occurring noble radioactive gas that poses significant health risks, particularly lung cancer, due to its colorless, odorless, and tasteless nature, which makes detection challenging without formal testing. It is found in soil, rock, and water, and it infiltrates indoor environments, necessitating regulatory standards and guidelines from organizations such as the Environmental Protection Agency, the World Health Organization, and the Occupational Health and Safety Agency to mitigate exposure. In this paper, we present various methods and instruments for radon assessment in occupational and environmental settings. Discussion on long- and short-term monitoring, including grab sampling, radon dosimetry, and continuous real-time monitoring, is provided. The comparative analysis of detection techniques—active versus passive—is highlighted from real-time data and long-term exposure assessment, including advances in sensor technology, data processing, and public awareness, to improve radon exposure evaluation techniques.

## 1. Introduction

Radon, a naturally occurring radioactive gas, poses a significant health risk when it accumulates in enclosed environments such as buildings or underground operations. It is a byproduct of the decay of radium, which, in turn, is a decay product of uranium-238. This colorless, odorless, and tasteless gas is challenging to detect without formal testing. Radon is commonly found in soil, rock, and water, generated from the decomposition of radium in these materials. As it seeps into buildings through cracks or openings, radon can accumulate at levels that are harmful to human health, particularly in basements and ground-floor constructions due to their proximity to the ground [1]. Outdoor environments, though they pose lower risks, can also exhibit elevated radon levels, especially in areas with high uranium content in the underlying geology [2]. Occupational settings such as mines and certain construction areas are known for potential radon exposure [3,4]. Exposure to high levels of radon is associated with an increased risk of developing lung cancer. When radon gas is inhaled, its radioactive progeny, particularly alpha particles from the decay of radon into polonium-210 in the uranium decay chain, can become trapped in the lungs and potentially damage lung tissue cells, leading to lung cancer [5]. Radon is thus a significant environmental and public health concern. Radon exposure is exacerbated in areas with poor ventilation, as the gas can accumulate to dangerous levels. Furthermore, radon levels can vary significantly from one location to another, making it crucial to conduct regular testing to ensure levels remain within safe limits.

Long-term exposure to elevated levels of radon has been linked to an increased risk of developing lung cancer, particularly in smokers [6]. The alpha particles emitted during the decay of radon and its progeny can damage lung tissue and DNA, leading to the development of cancerous cells [7]. This risk is further compounded by the fact that radon-induced lung cancer does not exhibit specific symptoms, making it difficult to detect until it has reached an advanced stage [8]. As such, mitigation strategies, such as improving ventilation and conducting regular radon testing, are essential for reducing the risk of exposure and protecting public health.

To address this concern, regulatory standards and guidelines have been established by organizations such as the Environmental Protection Agency (EPA), the World Health Organization (WHO), and the Occupational Safety and Health Administration (OSHA). These standards aim to protect the general public and workers from the harmful effects of radon exposure. The EPA, for example, has set the radon threshold in indoor air at 4 picocuries per liter (pCi/L) [9], while the WHO recommends exposure limits of 100 becquerels per cubic meter (100 Bq/m^3^) for indoor environments and 300 Bq/m^3^ for occupational settings [10]. OSHA has set a permissible limit of 1000 picocuries per liter (1000 pCi/L) for an 8-h time-weighted average in occupational settings [11]. In this paper, we describe methods and instruments used to assess radon levels in public environments and occupational settings.

## 2. Assessment Methods for Occupational and Environmental Radon Exposure

Various methods and techniques are available for assessing radon levels in both occupational and environmental settings. These settings require different approaches due to variations in anticipated radon concentrations and exposure durations.

### 2.1. Occupation Radon Methods

In occupational settings, radon assessment methods involve measuring radon concentrations in indoor workspaces and assessing worker exposure levels. Methods used include long-term monitoring, short-term monitoring, grab sampling, radon exposure modeling, radon dosimetry, continuous real-time monitoring, radon mapping, and indoor quality assessment.

Long-term and short-term monitoring involves the continuous measurement of radon levels over periods, ranging from several months to a year [12] and a few days for short-term monitoring [13,14], to capture average concentrations and identify fluctuations. Grab sampling, which collects air samples at specific intervals, is less commonly used in occupational settings due to its limitation in capturing variations in radon levels across the workday [15].

Radon exposure modeling uses computer simulation and mathematical models to estimate workers’ potential exposure to radon based on factors such as ventilation rates, building characteristics, and work activities. This method is used in addition to direct measurement methods [16]. Radon dosimetry involves workers wearing dosimeters to measure personal exposure to radon over a specific period [17], while continuous real-time monitoring uses automated devices to provide real-time measurements of radon concentration [18]. The most uncommon method is radon mapping, which involves creating a spatial map of radon concentrations in work or public environments [19]. Some studies combine indoor quality assessment with radon monitoring methods to measure radon concentration together with the presence of other indoor air pollutants, such as carbon dioxide, volatile organic compounds, and particulate matter [20].

#### Radon in Mining Settings

Radon exposure risks in mining settings are notably higher compared with general commercial and residential settings [21]. Miners in uranium, thorium, and ore mines are exposed to higher levels of radon progeny. These radioactive decay products can attach to lung tissue during inhalation, increasing the risk of lung cancer [21]. This elevated risk results from the levels of radon exposure and the duration of exposure. As such, the health implications of radon exposure in occupational settings are multifaceted, impacting the physical health of miners and health and safety regulations within the mining industry.

Kreuzer et al. [22] provide an understanding of the linear relationship between radon exposure and lung cancer. This insight emphasizes the unique occupational hazards miners face. In 2017, Daniels and Schubauer-Benrigan discussed the variance in radon exposure across different workplaces, stating the need for updated and enhanced measures in mining industries [23]. The scope is further broadened beyond uranium mines by Fan et al., advocating for tailored monitoring strategies for accurate assessment and radon protection [24]. These studies advocate for policy changes and enhancements in monitoring and regulatory practices.

### 2.2. Environmental Radon Methods

The investigation of radon assessment in environmental contexts is essential for understanding the potential health risks associated with radon exposure [1]. Given that radon is a primary cause of lung cancer in non-smokers [5], the importance of effective assessment strategies cannot be overstated. These strategies play a critical role in identifying areas with elevated radon levels and informing the development and implementation of necessary mitigation measures to safeguard public health.

Short-term monitoring, a method commonly used in environmental settings, involves the use of charcoal canisters or continuous radon monitors to measure radon concentrations over a period of 2–7 days [11]. Long-term monitoring, another method prevalent in environmental contexts, utilizes alpha track detectors or electret ion chambers to measure radon concentrations over a longer period, typically ranging from 3 months to 1 year [20]. Grab sampling, on the other hand, entails collecting air samples in containers such as activated charcoal canisters or grab sampling bags for a short period (minutes to a few hours) and then analyzing the samples in the laboratory to determine the radon concentration [25].

Soil gas monitoring is another important method involving the measurement of radon concentration in soil gas. This is achieved through techniques such as soil gas probe sampling or sub-slab sampling, followed by analysis of the samples for radon concentrations [26]. Additionally, building diagnostics employ various methods and instruments, including real-time monitoring, passive or active dosimeters, and radon mapping techniques, to assess radon levels within structures [15].

#### Retrospective Radon Dosimetry

Retrospective radon dosimetry is a technique used to assess past exposures to radon, especially in cases where real-time monitoring is not possible. This method finds application in historical exposure assessment, legal cases involving past radon exposure, and epidemiological studies [27]. It enables the assessment of lung cancer risk associated with residential radon exposure by analyzing long-lived radon decay products like polonium-210 (210Po) implanted in glass surfaces. This assessment facilitates the understanding and quantification of health risks from previous decades in epidemiological contexts [28]. In legal contexts, this technique supports legal evaluations by providing evidence of past radon exposure levels due to inadequate safety measures [27]. Additionally, retrospective radon dosimetry aids in historical exposure assessment in areas where monitoring was never implemented, assisting in the reconstruction of radon exposure levels over long periods. This reconstruction utilizes the alpha-emitting decay products of radon, embedded in various materials like glass, to estimate radon levels [29].

- Glass implantation method for retrospective radon dosimetry

The glass implantation method is significant in retrospective radon dosimetry. This method utilizes common glass items found in homes as natural detectors of radon decay products. Alpha particle tracks on glass surfaces are counted for radon assessment in indoor spaces [29].

This glass implantation method relies on the use of external track detectors that are temporarily fixed to the glass surface. Alpha particles from radon decay products such as polonium-210 impact these glass surfaces, not the glass itself. After exposure, it is the detector that is etched to reveal the tracks caused by these alpha particles, allowing for analysis of historical radon presence and concentrations. This is non-destructive to the glass, as it is never etched or altered during the process [29].

Optical or electron microscopes are used to analyze glass items, allowing for the precise counting and analysis of the alpha tracks on the glass surfaces. The historical reconstruction of radon exposure levels is achieved by estimating the density and distribution of the damage tracks [30].

This method was discussed and detailed in the seminal study published in *Nature* 334, in 1988, pp. 338–340, which demonstrated the utility of glass as a retrospective radon dosimeter [31]. The study provided valuable insight into long-term radon exposure levels in indoor environments, confirming that glass reliably records radon exposure levels. The glass implantation method remains a crucial tool, offering insights into past environments and exposures that would otherwise go undetected.

- CD/DVD method for retrospective radon dosimetry

This method utilizes common digital storage media, mainly Compact Discs (CDs) and Digital Versatile Discs (DVDs), as more innovative ways of measuring radon exposure. The polycarbonate layers in these discs react to alpha particles from radon decay, making them useful for radon radiation measurement [32].

The polycarbonate plastic and reflective metal used for data storage also capture alpha particles from radon decay products, leaving tracks similar to glass.

The analysis of radon exposure on discs involves subjecting them to a chemical etching process. This process entails treating the polycarbonate layer with a chemical solution that selectively erodes material along the paths damaged by the alpha particles, thereby revealing the tracks in a pronounced manner. These tracks are then examined under a microscope for a quantifiable count and measurement [33].

This method stands as innovative due to the use of abundant and low-cost materials readily available in most settings, both residential and office [34].

- Comparative analysis of the glass implantation method and the CD/DVD method

Retrospective methods such as the glass implantation and CD/DVD methods provide critical data for assessing historical radon exposures. Each method has its own unique advantages, limitations, and ideal conditions for use. Table 1 below presents a comparative analysis of the two methods. The choice between these methods depends on the specific requirements of the study, available resources, precision needs, and the environmental context [35]. 

## 3. Comparative Analysis of Radon Detection Techniques

Choosing the most appropriate radon detection technique depends on specific needs and circumstances. One of the primary considerations is whether to use an active or passive technique. 

Active techniques include devices like Continuous Alpha Radon Monitors (CARMs) and Electro-Static Precipitation (ESP) detectors (Table 2), which are ideal for short-term studies due to their real-time data and high sensitivity [37]. However, they are more complex and costly to operate.

Passive techniques, such as charcoal canisters, alpha track detectors (ATDs), and Electret Ionization Chambers (EICs), are favored for long-term monitoring due to their simplicity and cost-effectiveness, although they typically offer delayed results and lower sensitivity [37]. 

## 4. Assessment Tools for Radon Exposure

Modern radon measuring techniques are commonly categorized as passive or active, each offering distinct advantages and limitations tailored to specific evaluation needs.

Studies comparing active and passive techniques have highlighted the accuracy and reliability of active techniques in various settings, such as educational institutions and homes [38]. Passive techniques, on the other hand, rely on diffusion and natural decay for measurement, offering long-term exposure assessments and simpler deployment without the need for a power supply. Examples include alpha track detectors (ATDs) and Electret Ionization Chambers (EICs) [39]. Charcoal canisters are widely used due to their simplicity and affordability, adsorbing radon gas passively for later quantification using gamma spectrometry or liquid scintillation counting, which is frequently used [39,40]. ATDs capture radon decay alpha particles on a sensitive film, providing good sensitivity and long-term integration [41]. EICs measure changes in ion mobility to provide integrated exposure data, offering low cost and ease of use but lower sensitivity compared with other methods [10]. Additionally, radon measuring techniques can be categorized based on measurement duration. Short-term measurements span a few days to weeks, are useful for examining seasonal changes or sharp shifts in radon levels, and can be conducted using CARMs or activated charcoal canisters. Long-term measurements, typically taking months to years, are suitable for assessing average exposure and compliance testing and can be conducted using charcoal canisters, ATDs, or EICs.

## 5. Accuracy and Reliability of Radon Assessment Tools

Accuracy and reliability can vary depending on specific tool models, calibration, and environmental conditions. Table 3 demonstrates the accuracy and reliability of each technique.

## 6. Empirical Evaluation of Radon Detection Methods

The comparative analysis of various radon detection methods is substantiated by actual sampling results. The results highlight the distinct advantages and limitations of both passive and active radon detection methods, as well as the context-specific suitability of short-term and long-term monitoring approaches.

1. Long-term monitoring results:

Alpha Track Detectors (ATDs): Long-term monitoring using ATDs over a year in residential settings has revealed an average concentration of 200 Bq/m^3^; these findings are consistent with those reported in other high-radon areas, providing reliable data over extended periods, crucial for assessing long-term exposure risks [12].

2. Short-term monitoring results:

Charcoal Canisters: Short-term measurements using charcoal canisters have shown radon concentrations varying from 150 to 600 Bq/m^3^. These variations are seen particularly in basements and ground floors, indicating the influence of building characteristics and ventilation on radon accumulation. These short-term methods are invaluable for initial assessments and in scenarios requiring quick results [13].

3. Continuous real-time monitoring results:

Continuous Alpha Radon Monitors (CARMs): Real-time monitoring with CARMs has indicated occasional spikes in radon levels of up to 800 Bq/m^3^ in specific environmental conditions such as storms. This method’s ability to capture real-time fluctuations is essential for settings where levels might change rapidly, such as during certain industrial processes or in geographical industrial processes [38].

4. Grab sampling results:

This technique has demonstrated radon concentrations ranging from 100 to 700 Bq/m^3^ at different times of the day. The significant intraday variability captured by grab sampling is more relevant in occupational health studies where worker exposure may vary with activity and time [25].

5. Comparative analysis of detection techniques:

Active vs. Passive Techniques: Comparative analysis has revealed that active methods like CARMs provide higher sensitivity and real-time data, which are advantageous in environments with expected rapid changes in radon levels. Passive methods such as ATDs and EICs offer cost-effective solutions for long-term monitoring, suitable for residential radon risk assessment and compliance testing. These methods, though less sensitive, provide essential data for evaluating chronic exposure [37]. 

## 7. Limitations of Radon Detection Methods

In discussing the various methods of radon detection, it is important to acknowledge that the limitations of each method can affect the reliability and accuracy of the results. Table 4 below summarizes the limitations of radon detection methods.

## 8. Challenges in Assessing Radon in General Environments

Assessing radon in the environment presents various challenges due to the unique characteristics of radon and the variability of its sources and concentrations. Below are some of the primary challenges: 

Variability of Radon Concentrations: Radon concentration levels can vary extensively in various geographical areas and may also change over time in fluctuation in environmental factors such as humidity, temperature, and atmospheric pressure. This fluctuation means that a single measure is not representative of the exposure risk in a location; therefore, comprehensive testing is essential to accurately assess radon levels [48]. Detection and Measurement Techniques: Because radon is odorless, tasteless, and colorless, its detection requires specialized equipment, necessitating the use of sensitive and accurate detection devices [49]. Determining a suitable detection method is essential, as it can influence the reliability and accuracy of the measurements; also, the calibration of instruments and the standardization of measurement protocols are critical challenges [5]. Health Risk Assessment: The quantification of the health risks associated with cumulative radon exposure requires complex modeling, and this is further complicated by the need to consider individual susceptibility, lifestyle factors, and concurrent exposures to other indoor air contaminants [20]. 

Mitigation and Remediation: Radon mitigation, particularly in old structures, requires tailor-made site-specific solutions that include cost considerations while still remaining in compliance with safety standards and guidelines [50]. Public Awareness, Policy, and Financial Barriers: Public awareness of radon health risks and the necessity of testing and mitigation remain critical yet difficult. Radon testing and mitigation may be too expensive for individuals and organizations, requiring financial incentives or subsidies to promote widespread testing and mitigation. These issues demonstrate the need for combined education, regulation, and financial aid to decrease radon exposure risks. Radon management involves coordinated policymaking and implementation [50,51].

## 9. Best Practices in Radon Exposure Monitoring and Assessment

The implementation of best practices in radon exposure assessment and measurement is critical for accurately determining radon levels and effectively mitigating health risks. Several key practices are outlined below:

Utilization of Approved Measurement Devices: Radon measurement accuracy relies on instruments that adhere to national or international standards. Regulatory agencies, including the EPA (US), certify specific precision instruments. Short-term and long-term detectors are recommended based on assessment needs [10]. 

Strategic Placement of Radon Detectors: Proper detector placement is crucial for obtaining precise measurements. Detectors should be placed on the lowest occupied floor level, away from air currents, excessive humidity, and exterior walls, at a minimum height of two meters above the floor. This placement ensures measurements that accurately represent the radon levels occupants are primarily exposed to [52]. 

Long-term Testing for Accurate Exposure Assessment: Long-term testing for over 90 days provides a more precise representation of average annual radon exposure, considering seasonal variations. This approach offers a comprehensive perspective on long-term exposure [53].

Adherence to Closed-House Conditions: In short-term testing, maintaining closed-house conditions for at least 12 h before and during the testing period is essential. This minimizes the impact of external airflow exchange on radon concentration levels, enhancing measurement accuracy [39,53]. 

Repeat Testing and Professional Assessment: When results approach or exceed the threshold, repeat testing is advised to confirm the need for mitigation measures. In complex environmental contexts requiring comprehensive evaluation, engaging certified radon professionals is recommended [54,55]. Application of Quality Assurance and Control: Quality assurance measures and control procedures, such as duplicate testing and spiked samples, can ensure the reliability of radon measurements [56].

Compliance with National Guidelines and Standards: Adherence to guidelines and standards from health and environmental agencies ensures that radon testing and mitigation efforts align with best practices [5]. 

Educational Initiatives: Public health initiatives should educate building residents about the hazards of radon and the importance of conducting tests and implementing mitigation measures. Awareness campaigns can promote sound decision-making and encourage proactive measures [51]. 

Mitigation and Post-Mitigation Testing: Following the implementation of mitigation measures, conducting post-mitigation testing is crucial to verify the effectiveness of interventions in reducing radon levels.

## 10. Recommendations for Improving Radon Exposure Monitoring 

Enhancing radon exposure evaluation techniques is crucial for improving assessment accuracy and the efficacy of mitigation strategies. Several key areas for improvement include the following:

Advancements in Sensor Technology: Efforts to develop low-cost, highly sensitive, and portable sensors are underway. Current active techniques, such as Continuous Alpha Radon Monitors (CARMs), offer high sensitivity but are costly and complex. Research focusing on miniaturization, material innovations, and cost reduction could make these sensors more accessible for wider field applications. Additionally, exploring emerging sensor technologies, such as biosensors that use biomolecules sensitive to radon, shows promise for low-cost, real-time detection [57,58]. However, further research and validation are needed for their widespread adoption. Improving Data Processing and Analysis: The development of automated analysis tools for passive detectors, such as alpha track detectors (ATDs) and Electret Ionization Chambers (EICs), could streamline analysis and improve accuracy [59]. The manual analysis of these detectors can be time-consuming and subjective. 

The integration of real-time monitoring with data analytics using artificial intelligence (AI) algorithms could help identify temporal trends and potential sources and predict exposure fluctuations, offering deeper insights. Standardizing and harmonizing data storage and sharing by establishing standardized data formats and sharing platforms would facilitate data analysis across studies and regions, enabling broader assessments and risk prediction. Accessibility and Public Awareness: Efforts to reduce the cost of assessment tools and services are crucial, as high costs associated with some techniques prevent their widespread adoption. Strategies such as government subsidies, innovative financing models, and open-source technology development could increase accessibility [54,56]. Investing in public education and awareness campaigns is essential for raising awareness about radon risks and the importance of assessment, encouraging individuals and communities to take preventive measures. Developing user-friendly tools and resources, such as mobile applications and online platforms, can provide easily accessible information and guidance on radon testing and mitigation strategies, empowering individuals to manage their exposure risks [60].

## 11. Conclusions

Detection and measurement are the main challenges in managing radon exposure due to its imperceptibility. Regulatory bodies such as the EPA, WHO, and OSHA have established limits to safeguard public and occupational health. The choice between active and passive detection methods for radon assessment involves trade-offs in cost, sensitivity, and suitability for different settings. While active techniques like Continuous Alpha Radon Monitors and Electrostatic Precipitation Detectors offer real-time data with high sensitivity, they are costly and complex. In contrast, passive devices such as charcoal canisters and alpha track detectors are more affordable but less sensitive and require longer testing periods for results [61]. The variability of radon concentration levels due to geographical and environmental factors necessitates an integrated approach to radon assessment, highlighting the importance of long-term monitoring for accurate exposure risk assessment. Mitigating radon risks, especially in older buildings, requires tailored, cost-effective solutions that comply with safety standards. Public awareness and policy interventions are essential to increasing testing and mitigation efforts, underscoring the need for public education, regulatory control, and financial support to mitigate potential risks from radon exposure [39].

Future advancements in radon exposure assessment should focus on developing low-cost, highly sensitive, and portable sensor designs to enhance accessibility. Emerging sensor technologies with high sensitivity in passive detectors have the potential to revolutionize radon detection and risk assessment. Additionally, integrating real-time monitoring and analytics could significantly improve the accuracy and efficiency of radon measurement, along with the development of automated tools for passive detector analysis [10].

## Figures and Tables

**Table 1:** Comparative analysis of the glass implantation method and the CD/DVD method.

Criteria	Glass Implantation Method	CD/DVD Method
Accuracy	Accuracy varies; generally, within a factor of 2–3 at best by an order of magnitude.	Comparatively better accuracy is observed, with less variability in results.
Ease of Implementation	Requires access to specific types of glass and specialized microscopic equipment; can be challenging to implement widely.	Easier implementation due to widespread availability and affordability of CDs/DVDs and simpler analysis process.
Potential Limitations	Requires historical glass that has been undisturbed.	Quality and age of CD/DVD may vary, and environmental conditions may affect track preservation. Outdated in technology.
Preferable Conditions	Ideal for detailed studies in historical buildings or long-occupied residential homes where glass has not been disturbed.	Suitable for broad and cost-effective screening in residential and office environments. This method is good for preliminary assessments.
Contributions to the Field	Provides highly reliable data for detailed exposure assessment and scientific studies.	Facilitates widespread, basic radon exposure assessments.
Reference(s)	[29,31,36]	[34,35]

**Table 2:** Radon-specific techniques and comparison.

Technique		Advantages	Disadvantages	Cost	Sensitivity	Measurement Duration	Ideal for
**Alpha spectrometry of radon progeny (CARMs)**		Real-time data; high sensitivity	Expensive; complex setup; power required	High	Highest	Days to weeks	Short-term studies; source identification
**ESP detectors—concentrate radon for alpha spectrometry**		Highest sensitivity	Expensive; complex; power required	Highest	Highest	Days to weeks	Research; specialized applications
**Charcoal canisters—adsorb radon for laboratory analysis**		Simple; inexpensive	Requires lab analysis; delayed results	Low	Moderate	Days to weeks	Short-term sampling
**ATDs—record alpha particle tracks**		Long-term integration; easy deployment	Requires specialized analysis; delayed results	Moderate	Moderate	Months to years	Long-term monitoring; screening
**EICs**	Measure ion mobility changes due to radon	Easy to use; low cost	Lower sensitivity than others	Low	Moderate	Months to years	Long-term monitoring; screening

**Table 3:** Accuracy and reliability of radon assessment tools.

Technique	Accuracy	Reliability	References
**Continuous Alpha Radon Monitors (Carms)**	- High sensitivity (0.2 and 4 Bq/m^3^).	- Requires complex setup and power source.	[42]
**Electrostatic Precipitation (Esp) Detectors**	- Highest sensitivity (exceeds CARMs).	- Limited field applications due to complexity and cost.	[5]
**Charcoal Canisters**	- Moderate sensitivity (3–10 pCi/L).	- Standardized method with good reliability.	[39]
**Alpha Track Detectors (Atds)**	- Moderate sensitivity; varies with brand and type.	- Generally reliable for long-term monitoring.	[40]
**Electret Ionization Chambers (Eics)**	- Moderate sensitivity; varies with model and calibration.	- Reliability can be affected by environmental factors.	[10]

**Table 4:** Limitations of radon detection methods.

Detection Method	Limitation	Impact	References
Charcoal Canisters	Sensitivity to humidity	Humidity can saturate the charcoal, reducing adsorption and leading to underestimation.	[42,43]
Charcoal Canisters	Temperature-dependence	Temperature changes affect the diffusion rate of radon and its adsorption by charcoal, potentially affecting the results.	[43,44]
Electret Ion Chambers	Requires calibration	Sensitivity to static charges, thus requiring frequent recalibration.	[45]
Alpha Track Detectors	Long integration time	Though they have the ability to detect cumulative exposure, alpha track detectors offer no insight into short-term fluctuations or sudden radon increases.	[46]
Continuous Radon Monitors	High cost	Excessive costs limit accessibility for widespread residential use and routine testing.	[47]
Continuous Radon Monitors	Power dependency	Reliant on continuous power supplies, which can be limited in areas with power issues.	[47]

## Data Availability

Data are contained within the article.
